# Analyzing data from the digital healthcare exchange platform for surveillance of antibiotic prescriptions in primary care in urban Kenya: A mixed-methods study

**DOI:** 10.1371/journal.pone.0222651

**Published:** 2019-09-26

**Authors:** Legese A. Mekuria, Tobias FR de Wit, Nicole Spieker, Ramona Koech, Robert Nyarango, Stanley Ndwiga, Christine J. Fenenga, Alice Ogink, Constance Schultsz, Anja van’t Hoog

**Affiliations:** 1 Amsterdam Institute for Global Health and Development (AIGHD), Amsterdam, The Netherlands; 2 Amsterdam University Medical Centers, Location AMC, Meibergdreef, Amsterdam, The Netherlands; 3 PharmAccess Foundation, Amsterdam, The Netherlands; 4 PharmAccess Foundation, Nairobi, Kenya; 5 Gertrude’s Children’s Hospital, Nairobi, Kenya; Makerere University, UGANDA

## Abstract

**Background:**

Knowledge of antibiotic prescription practices in low- and middle-income countries is limited due to a lack of adequate surveillance systems.

**Objective:**

To assess the prescription of antibiotics for the treatment of acute respiratory tract infections (ARIs) in primary care.

**Method:**

An explanatory sequential mixed-methods study was conducted in 4 private not-for-profit outreach clinics located in slum areas in Nairobi, Kenya. Claims data of patients who received healthcare between April 1 and December 27, 2016 were collected in real-time through a mobile telephone-based healthcare data and payment exchange platform (branded as M-TIBA). These data were used to calculate the percentage of ARIs for which antibiotics were prescribed. In-depth interviews were conducted among 12 clinicians and 17 patients to explain the quantitative results.

**Results:**

A total of 49,098 individuals were registered onto the platform, which allowed them to access healthcare at the study clinics through M-TIBA. For 36,210 clinic visits by 21,913 patients, 45,706 diagnoses and 85,484 medication prescriptions were recorded. ARIs were the most common diagnoses (17,739; 38.8%), and antibiotics were the most frequently prescribed medications (21,870; 25.6%). For 78.5% (95% CI: 77.9%, 79.1%) of ARI diagnoses, antibiotics were prescribed, most commonly amoxicillin (45%; 95% CI: 44.1%, 45.8%). These relatively high levels of prescription were explained by high patient load, clinician and patient perceptions that clinicians should prescribe, lack of access to laboratory tests, offloading near-expiry drugs, absence of policy and surveillance, and the use of treatment guidelines that are not up-to-date. Clinicians in contrast reported to strictly follow the Kenyan treatment guidelines.

**Conclusion:**

This study showed successful quantification of antibiotic prescription and the prescribing pattern using real-world data collected through M-TIBA in private not-for-profit clinics in Nairobi.

## Introduction

Antimicrobial resistance (AMR) is recognized as a complex, multi-causal and serious public health threat of both medical and economic concern [1, 2]. According to the World Health Organization (WHO) and the US Centers for Disease Control (CDC), the inappropriate use of antibiotics in human and veterinary medicine, and in agriculture and livestock is among the most important cause for resistance development and spread [3, 4]. “Inappropriate” use may refer to the use of antibiotics while it is not necessary, the use of antibiotics that are inactive against the main relevant pathogens, the exposure to inadequate drug concentration that is not sufficient to kill bacteria, or exposure to drugs with an unnecessary broad spectrum of activity [5]. Several studies have indicated a linear and positive relationship between outpatient antibiotics consumption and resistance to that antibiotic [6, 7].

Antibiotics are among the most frequently prescribed groups of drugs in primary care [8, 9], and up to 50% of antibiotic prescriptions are believed to be unnecessary [1, 6]. Acute respiratory tract infections (ARIs) are among the leading causes for outpatient clinic visits [10, 11], with majority of them treated with antibiotics even though these are often self-limiting viral infections [12, 13]. Patient-, clinician-, and healthcare delivery- or policy-related factors collectively contribute to the inappropriate prescription of antibiotics [14]. According to recent analyses for Kenya and other low- and middle-income countries (LMIC), inappropriate antibiotics use is more frequent in LMICs than in high-income countries [15].

Previous studies about the use of antibiotics in ARIs in LMICs were undertaken mainly in public facilities where data sources are commonly paper-based patient records that are neither personalized nor real-time, and usually incomplete [16]. Patient-level prescription data in LMICs outside of the public sector are scarce [16]. Recent advances in mobile phone technology are rapidly entering the health service delivery system (collectively called mHealth or eHealth applications). mHealth applications have brought multiple opportunities to digitalize patient records, to communicate healthcare information, to channel payments and to facilitate billing practices [17, 18]. They may prove useful to strengthen antimicrobial stewardship in the private healthcare setting as most LMICs lack prescription monitoring and surveillance systems in primary care [19].

To obtain a more complete and dynamic picture of antibiotics prescription practices in primary care, we conducted an explanatory sequential mixed-methods study in which patient-level claims data generated in a digital healthcare exchange platform [20] were analyzed. Qualitative interviews were conducted to explain the quantitative results and to understand the views and experiences of clinicians and patients towards antibiotics use in ARIs.

## Methods

### Study setting

Between November 2016 and February 2017, we conducted a study in 4 outreach primary care clinics in Nairobi, Kenya, that are administered by a Charity Foundation [S1 Text]. All payments were channeled electronically through a digital healthcare data and payment exchange platform that enables people to open a ‘health wallet’ (branded as M-TIBA) on their mobile telephone in which they can save, receive, transfer, insure or pay money for healthcare. MTIBA connects people with a network of contracted providers [S1 Text].

At the time of study, nearly 50,000 people living in Nairobi slums had access to a fully donor subsidized health wallet that allowed them to access basic primary healthcare. Patient information related to clinic consultations, medical procedures or treatment, diagnoses and all the associated costs of medical care were collected through the digital claims system, providing digital records of healthcare transactions for each individual patient that can be tracked over time. We used this information for a quantitative analysis of antibiotic prescription. For a more in-depth understanding of the practices related to antibiotic prescription, we conducted an explanatory qualitative research in which healthcare workers and patients attending the clinics were interviewed.

### Ethical approval

The Ethical Review Board at the Gertrude’s Children’s Hospital in Nairobi, Kenya, approved the study protocol [REF: GCH/ERB/VOLMMXVI/100]. Clinicians and patients (guardians for patients aged <18 years) gave a written informed consent for qualitative interviews. Consent for claims data access was not required since patients had already agreed (when they signed-up to the M-TIBA wallet) that their data could be used if the General Data Protection Regulation (GDPR) compliance is met. To protect patients’ confidentiality and to keep their anonymity, we used de-identified patient codes.

### Quantitative/Claims data collection and analyses

Antibiotic prescription information was collected in real-time as part of routine clinical practice to claim payments for the healthcare provided by clinics. We used claims data of patients who received medical care between April 1 and December 27, 2016. Data of patients with the following ARI diagnoses were included: upper respiratory tract infections, influenza, rhinitis, sinusitis, acute otitis media, sore throat, tonsillitis, laryngitis, tracheitis, acute bronchitis or bronchiolitis, and pneumonia.

**Fig 1:**
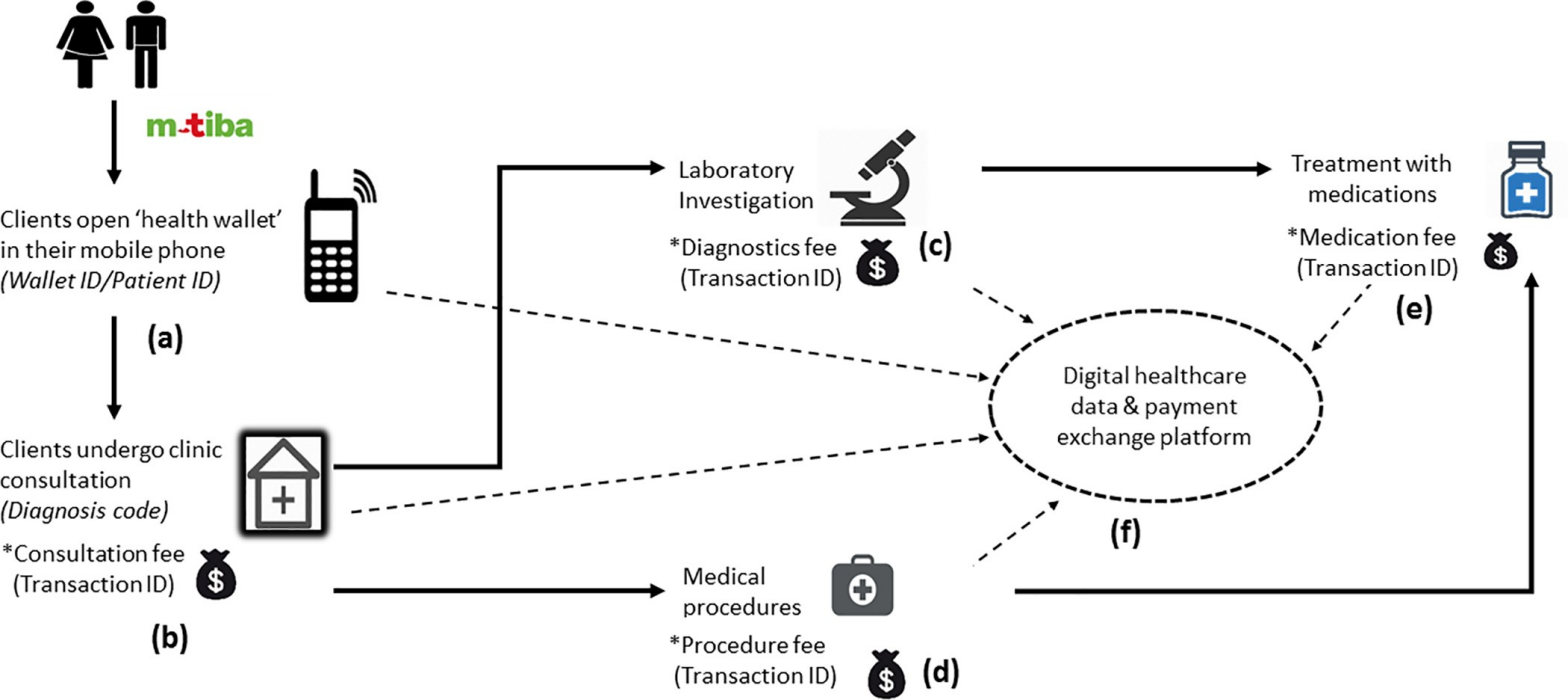
Diagram showing the digital healthcare exchange platform used to record patient information and to channel healthcare payments.

The claims data set consisted of three elements: user ID, diagnoses code/or description, and transaction/invoice ID for ‘items’ claimed [Fig 1—Diagram showing the digital healthcare exchange platform used to record patient information and to channel healthcare payments]. While user ID and patient ID are unique and remain constant, a new medical transaction/ invoice code is issued every time patients seek basic primary healthcare. Diagnoses coding was according to the international classification of primary care, second edition (ICPC-2) [21]. We used the medical transaction codes to record the number of clinic visits each person had made. We also used these codes to trace and count the number and types of medications dispensed per patient-visit. Because a patient could have multiple diagnoses, multiple medications prescribed per visit, and multiple clinic visits over time, the units of descriptive analyses were both patients and patient-visits. We used Excel 2016 and STATA 2012 [22] for claims data analyses.

### Qualitative data collection and analyses

#### Conceptual framework

We developed a deductive conceptual framework a priori [S1 Fig] based on previous literature [14, 23] and guided by results of the quantitative/claims data analysis. Three broad themes that could potentially determine antibiotics prescribing in ARIs were defined: clinician (provider)-related, patient-related, and healthcare delivery- or policy-related.

#### Interview guide and interviewers

Two interview guides were prepared: one for clinicians and one for patients [S2 Text]. Clinicians’ interview guide was based on deductive reasoning and contextualized to the Kenyan situation. It was prepared in English. Patients’ questions were first prepared in English, and translated to Kiswahili (the local language) by the fourth author, and back to English [24] by two people who could understand both languages. To assess the appropriateness and clarity of the questions, both clinician and patient interview guides were pilot tested in two of the study clinics one week ahead of the actual data collection date [24]. Two second-year Masters’ students at the University of Nairobi, and one clinical officer administered the interview guides after a one-day training of qualitative data collection [25] in preparation for this work.

#### Selection of interviewees and conduct of the interview

All clinicians who worked in the study clinics at time of study were interviewed at their practices. We used open questions to obtain their feedback on results of the quantitative/claims data analysis [S2 Text]. In addition, 1 specialist doctor, 1 general practitioner, and 1 clinical officer were interviewed to include the views and experiences of healthcare providers in private-for-profit facilities outside of the study clinics. The language of clinician interview was English.

Furthermore, 9 guardians of sick children (usually mothers) and 8 adult patients who were treated for ARIs at time of study were consecutively selected over 3 weeks and asked for consent to participate in the study. They were interviewed in Kiswahili through exit interviews outside of the clinics. Informal interviews were conducted with 4 lecturers at the School of Pharmacy, University of Nairobi, and with 2 officials working at the Ministry of Health.

### Qualitative data analyses

Patient interviews continued until the level of saturation. Interviews were audio-recorded and later transcribed verbatim. LAM and RK checked for the quality by listening to audio-records and looking over the transcripts simultaneously. Two persons who could understand Kiswahili and English translated transcripts back to English independently [24]. RK and another native speaker checked if the back translations were in agreement with the original Kiswahili versions.

We used Dedoose software version 7.0.23 for qualitative data entry and analyses [26]. After a thorough reading of full-length transcripts, LAM did the initial coding thematically based on pre-set codes according to the deductive conceptual framework [S1 Fig]. RK and AvH coded again 6 of the transcripts independently, and CF checked for consistency between the coding trees and for alignment with the conceptual framework. New codes or sub-codes emerging from the data collection and analyses, such as the influence of internet technology on patients’ demand for antibiotics and drug company relationship influencing clinicians’ antibiotic drug prescribing, were added to the coding tree. Excerpts that expressed similar thoughts or content were exported to an excel spreadsheet. These were linked to the corresponding pre-set codes, and were used to explain the quantitative results in the thematic analyses.

## Results

### Quantitative/Claims data analysis

The data collected through the digital platform allowed for analysis of patient-level clinical diagnoses and associated medication prescription (Tables 1 and 2).

**Table 1:** Characteristics of M-TIBA wallets, patients, patient-visits and diagnoses made at the study clinics between April 1 and December 27, 2016, in Nairobi, Kenya.

Characteristic	Value*
**M-TIBA wallets**	
Number of M-TIBA wallets signed-up (activated)	22,024
Number of activated M-TIBA wallets actually used	14,317 (65.0%)
**Patients**	
Total number of persons registered in M-TIBA wallets**	49,098
Number of females	30,163 (61.4%)
Age at registration—in years, n = 48,628	
≤ 18 years	25,515 (52.5%)
> 18 years	23,113 (47.5%)
Number of persons who ever visited an M-TIBA clinic	21,913 (44.6%)
Number of patients with two or more clinic visits	7,297 (33.3%)
Patient-visits***	
Total number of patient(clinic)-visits	36,210
Number of patient-visits with at least one patient-diagnosis	36,185 (99.9%)
Number of patient-visits per clinic	
Outreach clinic ‘A’	18,792 (51.9%)
Outreach clinic ‘B’	8,392 (23.2%)
Mobile clinic ‘C’	6,379 (17.6%)
Mobile clinic ‘D’	2,647 (7.3%)
**Patient-diagnoses**	
Total number of patient-diagnoses	45,706
Number of diagnoses per patient-visit, Range	1–5
Number of diagnoses per patient, Range	1–28
Number of patients with at least one diagnosis, n = 21,913	21,902 (99.9%)
Number of patients with:-	
No diagnoses at all	11 (0.1%)
Only one diagnosis	11,066 (50.5%)
Two diagnoses	5,646 (25.8%)
Three diagnoses	2,298 (10.5%)
Four diagnoses	1,148 (5.2%)
At least five diagnoses	1,744 (8.0%)

*Values are ‘n’ or n (%) unless otherwise indicated

** More than one person could be registered in a M-TIBA wallet.

*** Patient-visit refers to the number of clinic visits made by a person/patient for basic primary healthcare.

**Table 2:** Types of ARI diagnoses and disease-specific antibiotic prescription at the study clinics between April 1 and December 27, 2016 in Nairobi, Kenya (N = 17,739 ARIs).

**Categories of ARI diagnoses**	ICPC-2* **code**	**Frequency** **N** (%)**	**Antibiotics prescribed N (%)**
Acute upper respiratory tract infections	R74	7,041 (39.7%)	5,611 (79.7%)
(other) acute respiratory infections***	R83	4,425 (24.9%)	4,235 (95.7%)
Rhinitis	R97	2,337 (13.2%)	358 (15.3%)
Acute Tonsillitis	R76	1,351 (7.6%)	1,348 (99.8%)
Pneumonia	R81	946 (5.3%)	946 (100%)
Acute bronchitis/or bronchiolitis	R78	503 (2.8%)	318 (63.2%)
Influenza	R80	311 (1.8%)	192 (61.7%)
Otitis Media	H70—H72	291 (1.6%)	274 (94.2%)
Sinusitis	R75	151 (0.9%)	103 (68.2%)
(other) acute respiratory diseases****	R99	139 (0.8%)	131 (94.2%)
Streptococcal sore throat	R72	129 (0.7%)	111 (86.0%)
Sneezing/Nasal congestion	R07	99 (0.6%)	5 (5.1%)
Acute laryngitis/or tracheitis	R77	16 (0.1%)	14 (87.5%)

* ICPC-2 code: International classification of primary care, second edition

** A patient can have multiple clinic-visits or multiple diagnoses in a visit.

*** “R83—Respiratory infection, other” includes all other acute respiratory infections not classified nor included in R07, R71-

R78, and R80—R82.

**** “R99—Respiratory disease, other” is less specific; it is a ragbag class/code for all respiratory diseases not classified in the

other “R” classes (i.e., R01-R98).

A total of 22,024 M-TIBA wallets were signed-up through which 49,098 individuals were registered as clients. Of the 22,024 wallets, 8,204 (37.3%) had only one person registered. The median (inter-quartile range, IQR) number of persons in an M-TIBA wallet was two (1–3), with a maximum of 98. Of the 49,098 persons registered, 21,913 (44.6%) had at least 1 clinic visit, with a maximum of 24 visits. In total, 36,210 patient-visits were made, of which 18,792 (51.9%) were at Clinic ‘A’ (Table 1).

### Patient diagnoses

During the 36,210 clinic visits made by 21,913 patients 45,706 patient diagnoses were recorded. The number of diagnoses per patient-visit ranged from 1 to 5. The majority 27,362 (75.6%) of patient-visits had 1 diagnosis, 8,163 (22.6%) had 2, and 660 (1.8%) had 3 to 5 diagnoses. Twenty-five patient-visits (by 11 patients) had no diagnosis.

Overall, ARIs were the most frequent 17,739 (38.8%) patient diagnoses, followed by gastritis/or gastroenteritis 7,085 (15.5%), and skin diseases 3,796 (8.3%) (Table 3). Of the 17,739 ARI diagnoses, 7,041 (39.7%) were acute upper respiratory tract infections (Table 2). Furthermore, 357 (2.0%) ARI diagnoses were concurrent diagnoses of 2 different ARIs and 512 (2.9%) ARI diagnoses had at least one concurrent diagnosis other than ARIs. In summary: 16,870 of 17,739 (95%) ARI diagnoses contained one and only ARI diagnosis.

**Table 3:** Frequency of patient diagnoses at the study clinics between April 1 and December 27, 2016, in Nairobi, Kenya (N = 45,706 diagnoses).

Diagnoses description	Frequency	Percentage
Acute Respiratory Tract Infections (ARTIs)	17,739	38.8%
Gastritis/Gastroenteritis	7,085	15.5%
Skin Diseases	3,796	8.3%
Maternal and Child healthcare	3,238	7.1%
Urinary Tract Infection (UTI) syndrome	2,693	5.9%
Helminthes/parasitic worms	1,436	3.1%
Musculo-skeletal diseases	2,151	4.7%
Hypertensive Disorder/Cardiovascular Diseases (CVD)	1,287	2.8%
Diseases of Eye and Ear	1,194	2.6%
HIV/Malaria/Tuberculosis	1,159	2.5%
Sexually Transmitted Infections	922	2.0%
Neurological Diseases/Psychiatry	746	1.6%
Chronic Respiratory Tract Diseases	296	0.6%
Injury	190	0.4%
Diabetes	130	0.3%
Malignancy/Neoplasm	30	0.1%
Other diagnoses	1,614	3.5%

### Pattern of medication prescribing

A total of 85,484 medications were prescribed between April 1 and December 27, 2016 of which 21,870 (25.6%) were antibiotics, followed by anti-pains: 20,857 (24.4%) and antihistamines: 11,954 (14.0%) (Table 4). Amoxicillin constituted nearly one-third 7,061 (32.3%) of the total antibiotic drug prescriptions (Table 5).

**Table 4:** Types and frequency of medication prescriptions at the study clinics between April 1 and December 27, 2016, in Nairobi, Kenya (N = 85,484).

Types of medications prescribed	Frequency	Percentage
Antibiotics	21,870	25.6%
Anti-pains	20,857	24.4%
Antihistamines	11,954	14.0%
Medications for Gastritis/GIT problems	6,396	7.5%
Anthelminthic	3,922	4.6%
Antifungals/Topical ointments	5,607	6.6%
Vitamins/food supplements	4,701	5.5%
Anti-hypertensive drugs	2,231	2.6%
Steroids	1,965	2.3%
Eye/ear drops or ointments	1,613	1.9%
Medications for Asthma	1,477	1.7%
Anti-retroviral/Anti-TB drugs	925	1.1%
Other medications	1,966	2.3%

**Table 5:** Ranking the frequency of antibiotic drugs prescribed at the study clinics between April 1 and December 27, 2016, in Nairobi, Kenya (N = 21,870).

Type of antibiotic drug prescribed	Frequency	Percentage (%)
Amoxicillin	7,061	32.3%
Metronidazole	2,408	11.0%
Cefuroxime	2,386	10.9%
Azithromycin	2,005	9.2%
Co-trimoxazole	1,957	8.9%
Erythromycin	1,556	7.1%
Nitrofurantoin	913	4.2%
Ciprofloxacin	816	3.7%
Ampicillin/Cloxacillin	757	3.5%
Amoxicillin-clavulanic acid	605	2.8%
Flucloxacilin	595	2.7%
Norfloxacin	280	1.3%
Doxycycline	214	1.0%
Cefixime/Ceftriaxone	185	0.8%
Clarithromycin	67	0.3%
Penicillin Inject.	43	0.2%
Others	22	0.1%

### Treatment of ARIs with antibiotic drugs

Overall, 13,646 of 17,382 ARI diagnoses (78.5%; 95% CI: 77.9%, 79.1%) were treated with antibiotics between April 1 and December 27, 2016. Only 27 of 13,646 (0.2%) ARI diagnoses treated with antibiotics had a laboratory test of complete blood count (CBC). Of 7,762 children below 5 years of age and with single ARI diagnoses, 5,932(76.4%) were treated with antibiotic drugs. In adults above 18 years of age and with ARIs, antibiotic prescription was 83% (5,447 of 6,597). Because a patient could have multiple concurrent diagnoses that would require treatment with antibiotic drugs, we calculated the level of antibiotics use in patients with one and only ARI diagnoses. Accordingly, 13,195 of 16,870 (78.2%; 95% CI: 77.9%, 79.1%) ARI only diagnoses were treated with antibiotics between April1 and December27, 2016. Further analyses pertinent to potential differences in antibiotics prescription between genders and per time of day did not show a difference (results not shown).

Considering the choices of antibiotic drugs, 6,143 of 13,646 (45.0%) ARI diagnoses that were treated with antibiotics received amoxicillin, followed by azithromycin 1,699 (12.5%), cefuroxime 1,677 (12.3%), erythromycin 1,344 (9.8%), co-trimoxazole 1,151 (8.4%), amoxicillin-clavulanic acid 530 (3.9%), and other antibiotics 1,102 (8.0%) (Table 6).

**Table 6:** Ranking the frequency of antibiotic drug prescriptions for the treatment of ARIs at the study clinics between April 1 and December 27, 2016, in Nairobi, Kenya (N = 13,646).

Type of antibiotic drug prescribed	Frequency	Percentage (%)
Amoxicillin	6,143	45.0%
Azithromycin	1,699	12.5%
Cefuroxime	1,677	12.3%
Erythromycin	1,344	9.8%
Co-Trimoxazole	1,151	8.4%
Amoxicillin-clavulanic acid	530	3.9%
Metronidazole	399	2.9%
Ampicillin-Cloxacillin	278	2.0%
Nitrofurantoin	99	0.7%
Flucloxacilline	98	0.7%
Ciprofloxacin	75	0.5%
Ceftriaxone	59	0.4%
Penicillin Injection	21	0.2%
Doxycycline	20	0.1%
Clarithromycin	13	0.1%
Norfloxacin	13	0.1%
Other antibiotic drugs	27	0.2%

### Results of the qualitative interviews

Twelve clinicians (9 were working at the study clinics and 3 were working in private for-profit clinics outside of the study clinics) and 17 patients were interviewed about their views and experiences on antibiotics (over)prescribing or use for the treatment of ARIs. Various reasons that influence antibiotic (over-)prescribing in the primary care setting were mentioned (Table 7).

**Table 7:** Reasons mentioned for the excessive use of antibiotics to treat ARIs in 4 private-not-for-profit primary care facilities in Nairobi, Kenya, 2016.

Main Category	Sub-categories	Verbatim quotes
**1) Clinician (provider)-related**		
**1.1) Clinicians working at the 4 study clinics**		
	High patient-load with long queues	“Work load. They [clinicians] would just want to clear the long queue of patients [that] are too many, and want to prescribe, prescribe, and prescribe.” [Clinician 6, Clinical Nurse, Female]
	To keep patients away from hospital	“I think, we [clinicians] are doing a lot of unnecessary antibiotic prescription…a patient just comes in, and [we] do a review, and found no need to give antibiotics. But, [we] might decide to give just to keep patients away from [the] hospital” [Clinician 6, Clinical Nurse, Female]*
	To stock out ‘shortly’ expiring drugs	“[There is] even pushing drugs off because of short expiry” [Clinician 9, Clinical Officer, Female]
	Perception that clinicians must always prescribe	“I think, one is a culture that Doctors must prescribe a drug … many [clinicians] just believe that they [should] prescribe Amoxicillin, Paracetamol, or Piriton and Augmentin; that is when they [clinicians feel that they] have treated [the patient]” [Clinician 8, Clinical Officer, Male]
	Lack of knowledge	“The truth of the matter is that there is irrational use of antibiotics, which is a fact. I think much of it has got to do with the knowledge on the rational use of antibiotics, yes” [Clinician 1, Clinical Officer, Male]
	Not following a treatment guideline	“In some institutions, lack of protocol or failure to follow national guidelines” [Clinician 8, Clinical Officer, Male]
	Financial gain	“It’s obvious that we have over-prescription; people want to make profit. But, here at the [study] clinics, we have no such an encounter” [Clinician 5, Clinical Officer, Male]
	Clinicians ‘misled’ by parents’ claim that the sick child had previous exposure to antibiotics	“May be, a parent had bought Amoxicillin over-the-counter and had given to the [sick] child for 2 days; but, the parent [might] claim [that s/he] had given [the child] for 5 days, and did not improve. This might influence the clinician to give another antibiotic which may not warrant high potency than the first one” [Clinician 2, Clinical Nurse, F]
	Patients put pressure on the clinician	“Patients referred from other facilities say [that] they didn’t improve on the regimen they [had been given], but they cannot remember the drug, may put pressure on the clinician to put the patient on another antibiotic which could be more potent than the previous one”[Clinician 2, Clinical Nurse, Female]
	Frequent clinic visit by patients	“At times, it could be that you [the clinician] had seen the client, and it might be that the client come back again and you give an antibiotic which still might not be required” [Clinician 4, Clinical Officer, Male]
	Combining two antibiotics together to cover a wide range of micro-organisms	“If you don’t have a broad spectrum antibiotic, maybe you combine 2 antibiotics together you think they can offer the wide coverage, yes.” [Clinician 4, Clinical Officer, Male]
	Access to diagnostics	“Depending on whether the clinician has access to diagnostics, s/he may over-prescribe” [Clinician 5, Clinical Officer, Male]
	Clinicians’ perception that lab investigation may not always be necessary for ARIs	“Most of them [ARIs] don’t need anything else to be investigated in the laboratory unless its chronic, yeah” [Clinician2, Clinical Nurse, Female] “May not be very necessary; most of the time, i.e. 90%, we use history and physical examination, and 10% laboratory” [Clinician5, Clinical Officer, Male]
**1.2) Clinicians working outside of the study clinics**		
	Empirical treatment	“I think, many times it [antibiotics prescribing] is not evidence based. It is based on just empirical thinking.” [Clinician 10, Specialist Doctor, Male]
	“Absence” of treatment guideline	“We do not have guidelines for treating many infections, not just respiratory tract infections. . . Even if there are, they are not circulated widely enough.”[Clinician 10, Specialist Doctor, Male]
	No antibiotic stewardships in hospitals	“Not have good antibiotic stewardships in many hospitals. . . Some hospitals are trying to introduce stewardship. But, it is not spread wide enough, so people still prescribe the way they feel like” [Clinician 10, Specialist Doctor, Male]
	Fear that patient could deteriorate further	“Well, if I am over-prescribing, it is because of what I fear could be the sequel of this infection if it is not treated … I would fear that, may be, the patient could get worse.”[Clinician 10, Specialist Doctor, Male]
	Patient influence	“Sometimes, if patients have gone to a hospital, and they have not been given any medication, they feel like they were not treated. So, that could guide some clinicians in to prescribing unnecessarily” [Clinician 10, Specialist Doctor, Male] “[Some] patients say that I have come all this way, and paid all this money just to get some Paracetamol! Give me antibiotics”[Clinician 11, Medical Doctor, Female]
	Patient “safety”	“I think, it is more of better safety kind of thinking that you would rather over-treat than under-treat the patient” [Clinician 11, Medical Doctor, Female]
	Money first/To get more profit	“Especially in the private sector, no one is going to prescribe Amoxil. Because it is cheap. So, guys [clinicians] go straight to the Amoxil-Clavulanic combination … they tend to think about money first.”[Clinician 11, Medical Doctor, Female]
	Pressure from clinic owners	“When it comes to the private clinics, you also have other goals … you have target revenues. So, you are not going to prescribe drugs worth 100 Shillings, and yet you have the original drug worth 3,000 Shillings. So, you will tend to move towards the more expensive drugs, and keep away from the cheap drugs that are supposed to be used at first.” [Clinician 11, Medical Doctor, Female]
	Perceived “quality” of drugs	“They [clinicians] are trying to give quality drugs. [If] we do not have quality Amoxil, what we do have is quality Augmentin. So, you end up choosing drugs that you are not supposed to prescribe at first.”[Clinician 11, Medical Doctor, Female]
	Drug-company relationship	“Drug [company] representatives, usually come clinic to clinic, at least once or twice a week, and educate you on new drugs that are coming …If you feel that the drug is good for you, we normally just put in a request to the pharmacists to order more of it from the company.”[Clinician 11, Medical Doctor, Female]
	Diagnosis of ARIs based on clinical judgment	“You know, many times diagnosis of ARIs is based on the clinical judgment. It is just purely clinical judgment” [Clinician10, Specialist Doctor, Male]
	Kick out the disease at once	“You know, at times, the Doctors want to kick out the disease at once. They do not want another time [patient] visits. I think, that is the main cause [for over-prescribing]”[Clinician 12, Clinical Officer, Male]
	Fear that patients might not come again	'Yea, market. If a patient goes out, and tells others that you do not give them drugs, they would not come to you. So, if you give them, may be, an antibiotic for three days, and they take and feel okay, they always come.” [Clinician10, Specialist Doctor, Male]
**2) Patient-related**		
**2.1) As perceived by patients**		
	Patient expectations	“Yes. I had expected drugs so that my baby could recover [soon] … you wouldn’t come to a hospital if you were not given medicines” [Mother of Patient5, <5 years, Male patient] “I would have been shocked [if my son was not prescribed with antibiotics] because my son is ill … [would have] told him [the clinician] to examine him [my son] again”[Mother of Patient2, 1 year, Male patient]
	Patients might go to another clinic if they were not given medicines	“I would have looked for another place to be given drugs because of the way my baby is feeling and reacting.”[Mother of Patient5, <5 years, Male patient] “I would go to another hospital to seek for consultation [if I were not prescribed a medication]” [Patient13, 29 years, Male]
	Patients knew that healthcare was for “free”	“If I was paying, I wouldn’t have come because I don’t have money; however, I thought it was good that I came [here] because medicines are for free” [Patient9, 47 years, Female]
**2.2) As perceived by clinicians**		
	Patients insist on antibiotics	“Apart from the disappointment, some of them [patients] would insist or become violent, yes, they would insist, especially the old ones, they would insist you to give [them] antibiotics” [Clinician 1, Clinical Officer, Male]
		“[Some] patients just say: I have come all this way, and paid all this money just to get some Paracetamol! [Please] give me some antibiotics” [Clinician11, Medical Doctor, Female]
	Fear of patient disappointment	“One is that of disappointment. Yes, patients might feel disappointed and would air out their disappointment” [Clinician 1, Clinical Officer, Male]
	Patients’ “self-referral” from one clinic to another	“[patients] moving from one clinic to another and getting the same kind of treatment might end up with antibiotic over-prescription” [Clinician 3, Clinical Nurse, Female]
	Patients’ belief/perception	“They [patients] think that taking medication for all [diseases] will make them more healthy” [Clinician 3, Clinical Nurse, Female]
		“Most of them [patients] believe once they are sick they should always be given antibiotics”[Clinician4, Clinical Officer, Male]
	Patients influenced by internet technology	“Especially in the private sector, we get a lot of “google patients.” These are patients who already have the symptoms, and they run to google, they look at the symptoms, and they will come to you in a panic, and they will be the most annoyed when you do not give them antibiotics”[Clinician11, Medical Doctor, Female]
	Patients’ ‘self-referral’ to different clinics	“They have got one Doctor today, they are given, may be Augmentin, the child does not get better. They go the next day to another one, and they are given Zithromax, and they will go to another one.”[Clinician10, Specialist Doctor, Male]
	Patients will buy antibiotics anyway	“Even if you tell them [patients] that they are not supposed to take drugs, they will go to the shop [pharmacy] to buy it anyway” [Clinician12, Clinical Officer, Male]
	Patients having insurance	“Many times, it is the patients who have insurance who will demand a prescription because they are not paying for it themselves. The ones who are paying for it themselves do not even bother to go to the Doctor; they go to the pharmacy straight away and buy the medicine from there.”[Clinician10, Specialist Doctor, Male]
**3) Healthcare delivery- or policy-related**		
	Lack of treatment guideline	“We do not have guidelines for treating many infections, not just respiratory tract infections. . . Even if there are, they are not circulated widely enough.” [Clinician 10, Specialist Doctor, Male]
	No antibiotic stewardships in hospitals	“Not have good antibiotic stewardships in many hospitals. . . Some hospitals are trying to introduce stewardship. But, it is not spread wide enough, so people still prescribe the way they feel like” [Clinician 10, Specialist Doctor, Male]
	Access to diagnostic facilities	“Depending on whether the clinician has access to laboratory investigations, s/he may over-prescribe” [Clinician 5, Clinical Officer, Male]
	Absence of diagnostic facilities	'No, right now we don’t have any [lab investigations which are readily available to diagnose ARTIs]” [Clinician4, Clinical Officer, Male] “Well I don’t know any lab investigations which are readily available [to diagnose ARIs]” [Clinician2, Clinical Nurse, Female]

* In the study setting, Nurses with a BSc degree qualification are entitled to see patients and to prescribe medications at the primary care/OPD level

### Clinician-related factors

Clinicians at the study healthcare facilities had different views on their current prescription of antibiotics in ARIs. Some explained the “excessive” use of antibiotics to treat ARIs in the absence of strong medical indications.

“*I think*, *we are doing a lot of unnecessary antibiotic prescription” [Clinical nurse]*

However, other clinicians argued that their current use of antibiotics in ARIs was rational, and could be justified. They repeatedly mentioned their commitment to strictly follow the treatment guidelines.

“*Here at [the study clinics]*, *we follow guidelines and*, *in case of anything*, *we consult [seniors]” [Clinical officer]*

In contrast, two doctors from private-for-profit clinics said that they did not have to necessarily follow a clinical guideline to treat ARIs. They also said that they did not know if a treatment guideline for ARIs was available.

“*I do not think there is [a guideline]*. *Well*, *I do not follow any guidelines” [Specialist doctor]*

“*No*, *currently we do not have [a guideline]*, *and do not use it” [Medical doctor]*

#### Reasons for (over)prescription of antibiotics in ARIs

Clinicians reflected on their thoughts and experiences about the most sensible reasons behind the “excessive” prescription of antibiotics to treat ARIs. They mentioned various reasons related to patients, clinicians themselves, and the healthcare delivery system or policy at large. We summarized their responses in Table 7.

#### Choice of antibiotic drugs to treat ARIs

Considering the choice of antibiotics to treat ARIs, all clinicians at the study clinics said that they would prefer penicillins as the first-line drug to treat ARIs, which is in line with treatment guideline recommendations [27].

“*Following up the guidelines*, *I would always start with penicillins; the first line basically is amoxicillin” [Clinical officer]*

However, responses from clinicians working in private-for-profit clinics indicated the tendency to prescribe more broad spectrum and “expensive” antibiotics as a front-line to treat ARIs.

“*From my own experience*, *augmentin [amoxicillin-clavulanic acid] would work*. *If the infection is mild*, *azithromycin comes in” [Specialist doctor]*

### Patient-related factors

All the interviewed patients had received antibiotic prescriptions for the treatment of ARIs at the study clinics. Majority of them had expected medicines when they visited the clinic.

“*I had expected drugs so that [my] baby could recover [soon]” [Mother of a sick male child]*

When patients were asked whether they had put pressure on the clinician to give them antibiotic medications, almost all said that they had not.

“*Not really*! *The doctor is the one to use his own investigation…*. *He [the Doctor] knows what is best for his clientele*, *so I did not [have to put] pressure [on] the doctor” [Adult patient*, *female]*

#### Perceived disease severity

Patients were also asked how they would have reacted had the clinician decided not to prescribe them (or to their sick child) a medicine. Some said that they would have reacted immediately.

“*I would have been shocked because my son is too sick*. *[I] would have told him [the doctor] to examine him [the child] again” [Mother of a sick male child]*

Others said that they would have trusted the doctor, and would have accepted the doctor’s decision.

“*I would not have felt angry because he [the doctor] is the one who treats me*. *He [the doctor] knows what I should be taking” [Adult patient*, *male]*

Patient-related factors that influence antibiotics prescribing as perceived by the clinician are summarized in Table 7.

### Factors related to the healthcare delivery system or policy

#### Availability of laboratory tests to diagnose ARIs

All clinicians mentioned ARIs among the most common reasons for outpatient clinic visit. Many of them said that the diagnosis of ARIs is based primarily on clinical judgment.

“*You know*, *many times*, *it is based on the clinical judgment*. *It is just purely clinical judgment—it is getting the history*, *and just examining and just seeing the signs—that is it” [Specialist doctor]*

In addition, almost all clinicians at the study clinics reported that they lack laboratory investigation to diagnose or to rule-out the diagnoses of ARIs. Some of them also said that laboratory investigation might not be necessary for acute infections, such as ARIs.

“*I don’t think of laboratory investigation for acute [respiratory] infections*.*” [Clinical officer]*

In contrast, a female medical doctor who works in a private-for-profit clinic emphasized the need for laboratory investigations in ARIs to avoid misdiagnosis and the inappropriate use of antibiotics (Table 7).

“*I would definitely go for a full blood count to see Lymphocytosis*, *Leukocytosis … If they are increased*, *it is a marker of infection*, *and I would start antibiotics* …*” [Medical Doctor]*

#### National AMR policy and surveillance guide

At the time of study, we could not find a policy document to address AMR in Kenya, except a clinical guideline for the management and referral of common conditions (including ARIs) in primary care [27]. In this treatment guideline [27], the different categories of ARI diagnoses with the preferred choices of therapeutics are not explicitly described nor sufficiently addressed. The treatment guideline mentions indications for appropriate use of antibiotics when suspecting bacterial infections. It also describes in a sentence that “*antibiotics are of no value in acute upper respiratory tract infections*.*”*

## Discussion

In this study we quantified antibiotics prescribing in 4 private not-for-profit clinics using real-world data collected in a digital healthcare exchange platform (M-TIBA). Reasons for antibiotics (over)prescribing in the treatment of ARIs were also explored. We found substantial variation in antibiotics prescribing between different classes of ARIs, which is in agreement with previous study results [9, 28]. However, the antibiotic drugs chosen were generally those recommended for first-line treatment according to the national treatment guideline in Kenya [27]. This suggests that stewardship interventions [29] should focus primarily on reducing antibiotics usage in ARIs that are often treated with antibiotic drugs inappropriately.

Close to 80% of ARIs (with or without concurrent diagnoses) were found treated with antibiotics. The ICPC-2 diagnosed acute upper respiratory tract infections, (other) acute respiratory infections, and acute tonsillitis, which together constituted nearly three-fourth of all ARI diagnoses reached antibiotics prescription levels of up to 99.8%. These high degrees of prescription surpass levels recommended by national [27] and international [28, 30] standard treatment guidelines. Such clinical guidelines recommend ‘no’ antibiotics use in (non-specific) acute upper respiratory tract infections (such as the common cold and acute bronchitis) [27, 28, 30] or the ‘restricted use,’ i.e., <15%, of antibiotics in acute pharyngitis and rhino-sinusitis [28, 30] as majority of them are self-limiting viral infections and only a small percentage might be complicated by bacterial infections [12, 13, 28, 31]. Previous studies showed ‘no’ or ‘limited’ benefit of using antibiotics in these ARIs [28]. The observed antibiotics prescription level for ARIs in the current study is higher than previous study reports of 54% in the UK, 53% in South Africa, 48% in India, 38% in the Netherlands and 25% in other European countries [9, 32–35]. Our results are comparable with study findings in Swaziland and in China [36, 37].

The high level of antibiotics prescription found was partly explained through qualitative interviews and appeared due to a combination of clinician- and patient-related factors, including high patient load, clinician and patient perceptions that clinicians should prescribe, perceived demand and expectation from patients, and sometimes clinics want to offload near-expiry drugs (Table 7). Such findings are in agreement with results from previously conducted studies [38, 39]. An additional reason could be that existing national treatment guidelines in Kenya [27] are not sufficiently detailed to explicitly mention when and which antibiotic drugs to use for which categories of ARIs. It is recommended to update Kenyan treatment guidelines in general [27], since these are published nearly a decade ago. Another explanation for the high level of antibiotics prescription could be the absence of a national policy package for AMR which could frame and clearly outline the list of priority interventions needed to promote prudent antibiotics use. It was discussed in the informal interviews that a national AMR policy is underway and this has since been published [40].

The present study shows that the claims data collected in semi-real time digital healthcare exchange platform offers a unique opportunity to monitor the pattern of antibiotics (over-) prescription in relation to patient diagnoses (such as ARIs) at low costs. Using these data could allow monitoring at scale the prescription of antibiotics, and helped provide social-norm feedback timely to both healthcare staff and patients, in order to improve the quality of care [41]. In the long term, the real time, actionable data can help reduce the economic wastage of antibiotic over-prescription and concomitant threat of drug resistance.

Digital platforms could potentially integrate algorithms that represent national treatment guidelines, which can help clinicians to remind them of guideline recommendations for proper therapeutic decision making [42, 43]. In addition, the technology can help flag deviations in clinical or diagnostic decision making, e.g., by showing pop-up messages on the computer screen or through mobile phone Apps of both doctors and patients providing feedback in real-time [42, 43, 44]. Such features are currently developed through patient-trackers that help clinicians provide high quality services to patients.

Strengths of our study include the use of real world patient-level medical data collected in a digital healthcare exchange platform in real time. However, the data collected through the platform is limited to claims data, and does not store data on treatment outcomes or laboratory test results. The ability to link patients’ clinical diagnoses with treatment information helped to estimate the degree of antibiotics prescribing in ARIs. This estimate could be used as a baseline for future stewardship interventions. A limitation of quantitative claims data is that it does not fully capture the circumstances in actual clinical practice, such as reasons for antibiotics prescription. However, supplementary application of the qualitative method helped to validate findings.

We might over-estimate the degree of antibiotics prescribing since a patient could have multiple and concurrent diagnoses that require treatment with multiple antibiotic drugs. However, we re-calculated the level of antibiotic prescribing in ARI-only diagnoses without concurrent infections, and the two estimates are closely similar. The analysis of prescription data, as opposed to usage, might over-estimate the actual utilization of antibiotic drugs.

We did not validate the diagnoses of ARIs, as data were retrieved from an electronic repository retrospectively. In addition, uncertainty and misclassification of coding between the different categories of ARI diagnoses cannot be ruled out. This is because clinicians indicated that they had followed the Kenyan treatment guidelines that are not sufficiently detailed with respect to the specific ARI categories and the associated treatment recommendations. It should also be understood that the current study setting is not typical or representative of private clinics in Kenya because patients get “free” diagnosis and treatment as part of a social program administered by donors and a charity foundation. This might influence clinicians’ behavior towards generous prescription of antibiotics. Therefore, generalization of the study results should be considered cautiously.

In conclusion, results from the present study showed that the degree of antibiotics prescribing to treat ARIs in this primary care setting in Kenya is high. However, the types of antibiotic drugs prescribed are generally those recommended in national treatment guidelines. A combination of factors at all levels of the healthcare delivery system contributed to such high levels of antibiotics prescription. Digital platforms can help to timely collect patient-level data, and to support locally developed real-time treatment algorithms, clinical decision support systems, or auditing and feedback tools to constantly remind clinicians for judicious antibiotics prescribing in primary care practices. Ultimately, it improves the quality of care and significantly reduces the economic and public health burden of over-prescription.

## Supporting information

S1 Fig Deductive conceptual framework (for the qualitative component) based on previous literature and guided by results of the quantitative/claims data analysis. (TIF)

S1 Text M-TIBA used as a digital healthcare data and payment exchange platform at the Gertrude’s outreach and mobile clinics. (PDF)

S2 Text Open ended questions for clinicians and patients who participated in the present study. (PDF)

S1 File Dataset. (XLSX)
