# Correction: Analyzing data from the digital healthcare exchange platform for surveillance of antibiotic prescriptions in primary care in urban Kenya: A mixed-methods study

**DOI:** 10.1371/journal.pone.0225846

**Published:** 2019-11-21

**Authors:** Legese A. Mekuria, Tobias F. Rinke de Wit, Nicole Spieker, Ramona Koech, Robert Nyarango, Stanley Ndwiga, Christine J. Fenenga, Alice Ogink, Constance Schultsz, Anja van’t Hoog

The second author’s name appears incorrectly in the author list, citation, and Author Contributions. The correct name is Tobias F. Rinke de Wit. The correct citation is: Mekuria LA, Rinke de Wit TF, Spieker N, Koech R, Nyarango R, Ndwiga S, et al. (2019) Analyzing data from the digital healthcare exchange platform for surveillance of antibiotic prescriptions in primary care in urban Kenya: A mixed-methods study. PLoS ONE 14(9): e0222651. https://doi.org/10.1371/journal.pone.0222651
