# Supplementary figures and images for: Analyzing data from the digital healthcare exchange platform for surveillance of antibiotic prescriptions in primary care in urban Kenya: A mixed-methods study
Source: PLoS One. 2019 Sep 26;14(9):e0222651. doi: 10.1371/journal.pone.0222651 (PMC6762089; doi:10.1371/journal.pone.0222651)

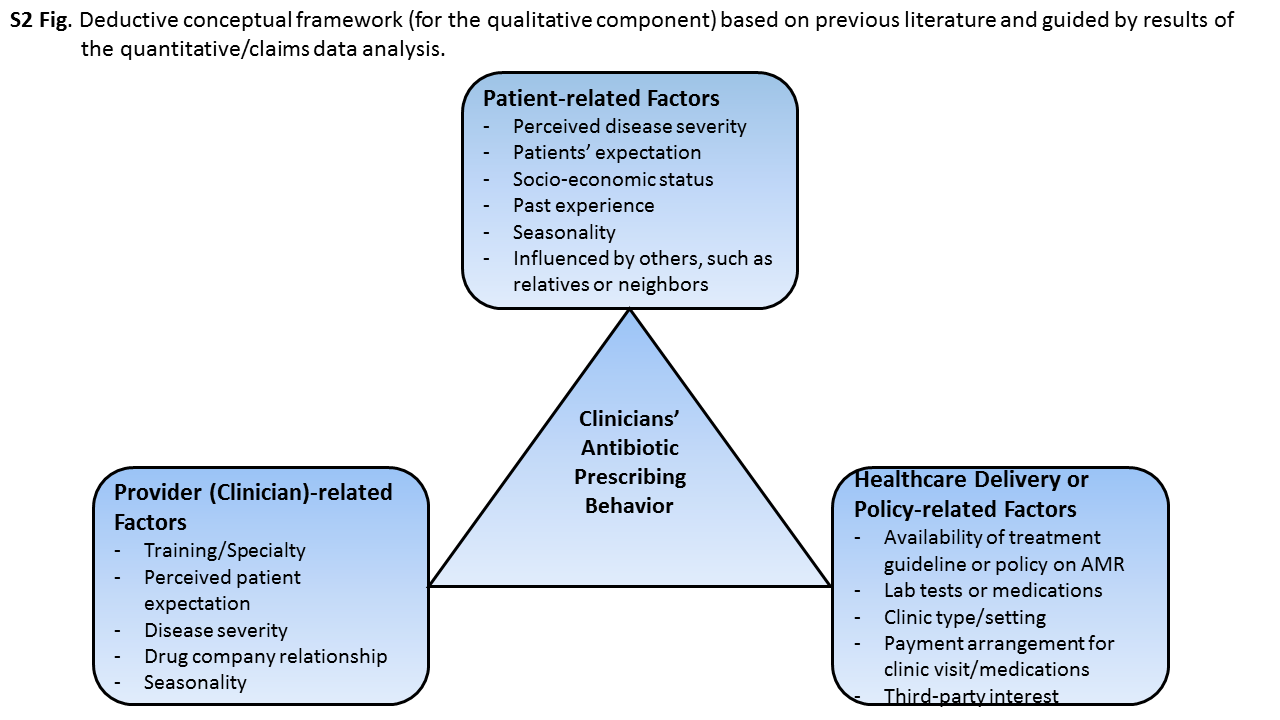

Supplement: S1 Fig — (TIF) [file pone.0222651.s001.tif]
