# Supplementary material for: Analyzing data from the digital healthcare exchange platform for surveillance of antibiotic prescriptions in primary care in urban Kenya: A mixed-methods study
Source: PLoS One. 2019 Sep 26;14(9):e0222651. doi: 10.1371/journal.pone.0222651 (PMC6762089; doi:10.1371/journal.pone.0222651)
Supplement: S1 Text — (PDF) [file pone.0222651.s002.pdf]

Supplementary File 1

M-TIBA used as a digital healthcare data and payment exchange platform at the Gertrude's outreach and mobile clinics

This study was conducted in 4 primary care clinics in Nairobi, Kenya: Githogoro, Mathare, Kosovo, and Kiambu. They are outreach or mobile clinics, as part of a program administered by the Gertrude's Foundation to provide free healthcare to poor people inhabiting the Nairobi slums [1]. All healthcare payments were channeled through M-TIBA ('M' for mobile phone and 'TIBA' means healthcare in Swahili). M-TIBA is a mobile telephone-based healthcare data and payment exchange platform, which was rolled out by CarePay Ltd, Kenya in partnership with Safaricom (a local telecom operating company) [2]. The platform connects people with providers working at the contracted clinics, and enables them to open "health wallet" on their mobile telephone in which they can save, receive, transfer, insure or pay money for healthcare. CarePay administers healthcare payments, and processes claims data on M-TIBA [3]. M-TIBA also allows pooling donor funds to provide free or subsidized medical care to poor people.

In the study clinics, a program called Smiles was built on M-TIBA with the financial support from Gilead Foundation [1]. At the time of the study, nearly 50,000 poor people living in Nairobi city received free access to basic primary healthcare. All healthcare payments related to consultation, diagnostics, medical procedures, or treatment were channeled through M-TIBA, providing digital records of all patient diagnoses. A typical patient trajectory in the Gertrude's Smiles clinic is as follows. First, people with Safaricom telephone line open a Smiles healthcare wallet in their mobile phone with the help of an "mTIBA agent". Up on registration, they receive a unique User Wallet ID, which shall never be shared with other people except with dependents (such as spouses or children) who could register as secondary beneficiaries. Next, each person registered in the Smiles health care wallet gets a unique patient ID at clinic reception. Then, patients get a new card (or booklet) in which clinicians record patients' medical information manually. After completion of medical care, a unique medical transaction code is issued for the types of health care patients had received, such as consultation, medical procedures, or treatments given. In the next patient encounter, a new medical transaction code will be issued again. Finally, patients' diagnostic, treatment and medical transaction information is recorded electronically in the pharmacy at the time of medication dispensing. CarePay automatically receives an electronic copy of the pharmacy records for claims assessment and payment approval [3].

References

1. https://www.gerties.org/news/news/gilead-foudnation-and-pharmaccess-foundation-partners-with-us.
2. https://www.pharmaccess.org/update/safaricom-launches-transformative-partnership-to-promote-healthcare-inclusion-in-kenya/.
3. https://carepay.co.ke/product/m-tiba
