# Supplementary material for: Analyzing data from the digital healthcare exchange platform for surveillance of antibiotic prescriptions in primary care in urban Kenya: A mixed-methods study
Source: PLoS One. 2019 Sep 26;14(9):e0222651. doi: 10.1371/journal.pone.0222651 (PMC6762089; doi:10.1371/journal.pone.0222651)
Supplement: S2 Text — (PDF) [file pone.0222651.s003.pdf]

Supplementary File3

A) Open-ended questions for clinicians

INTRODUCTION

Hello! My name is ……………… and I work for the PharmAccess Foundation, SafeCare/M-TIBA program in Nairobi, Kenya.

I would like to have a short discussion with you about medication use during an acute respiratory tract infection. I will interview about 25 people in various communities around Nairobi. The results of this interview will be used to improve the quality of healthcare in these and other similar clinics here in Nairobi.

Let me assure you that the information you tell me now will only be used for healthcare quality improvement in the mTIBA clinics. Please, feel free to ask questions at any time during or after the interview. There is no right or wrong answer to these interview questions. If there are issues which you do not like to talk about, then let me know.

Would you like to participate in this short interview? 1) Yes 2) No

Interviewer: If replied “YES”, then thank the patient, ensure a written informed consent has been made, and start the interview.

1) **Let’s start by talking about your field of training/specialty and employment status**
   - Can you tell me your field of training/or specialty?

     ***Probe**: Medical Doctor, Specialist Doctor, Clinical Officer, Nurse, …*
   - Are you working as an employee or running your own clinic?
   - What kinds of health services does the clinic provide?

     ***Probe**: patient consultation, lab investigation, selling drugs, …*
2) **Now, let’s talk about the common diagnoses in the outpatient clinic**
   - Based on your experience, which disease conditions are commonly seen in the outpatient clinic/primary care? **Probe**: ARTIs, …
   - What about the standard national treatment guideline to manage ARTIs? (**Probe**: is it available, are you using it, etc . . .)
   - How would you describe your clinical practice of treating ARTIs in relation to strictly following the national treatment guideline?

3) **Great! Let's move on to the diagnosis and clinical management of acute respiratory tract infections (ARTIs)**

- Which sets of diseases/or health problems would you classify as an Acute Respiratory Tract Infection (ARTI)?
- Suppose a patient with signs and symptoms of an ARTI comes to your clinic. Can you explain to me the series of steps you would follow to diagnose & treat him/her well? ***(Probe**: whether it is clinically, by doing a physical examination, or doing a laboratory investigations*)
- Can you tell me about any laboratory investigations which are readily available and used in your clinical practice to help you to diagnose ARTIs?
- Can you explain to me how you would differentiate between ARTIs of bacterial and viral etiology?
- What is your opinion towards the use of a rapid and affordable diagnostic test (*such as serum CRP test*) to help to differentiate between ARTIs of bacterial and viral etiology?

4) **Let's continue our discussion about the treatment of ARTIs**

- Can you tell me what kinds of medications you would prescribe to a patient presenting with signs and symptoms of ARTIs?
- Can you explain to me when you would prescribe an antibiotic to a patient diagnosed as having an ARTI? Would you wait for the laboratory investigation results before you started prescribing an antibiotic?

5) **We are now half way in our discussion**

- Let's assume that you prescribed an antibiotic to a patient diagnosed as having an ARTI. Can you tell me something about the availability of these drugs? What happens if a patient can't find the drug in a clinic? Go to a pharmacist/chemist?
- Can you tell me who usually pays for health care utilization (consultation, lab investigations, and medications) among patients diagnosed as having ARTIs?
- Can you say something about the affordability of health care, including the drugs?
- What is your opinion about the possibility that patients with signs and symptoms of ARTIs getting access to OTC drugs by themselves (i.e. without a prescription)?

6) It is now time for discussion about antibiotics prescribing & usage

- Can you tell me which antibiotics would you prescribe to a patient diagnosed as having an ARTI?
- Which aspects of the patient would you consider before you prescribed an antibiotic drug to a patient (with an ARTI)? ***Probe:*** *socio-economic status, disease severity, patient expectation, payer characteristic, …*
- Imagine that a patient with an ARTI comes to you and demands an antibiotic while you think it is not necessary. What would you do then? Is it something that occurs frequently? How frequent it is: 1 in 10?
- In your opinion, what additional factors you might think put pressure on the clinician to frequently prescribe antibiotics despite they might not always be necessary to treat ARTIs? ***Probe:*** *patients themselves? employer? Pharmaceutical companies? Colleagues? Treatment guidelines?*
- Imagine, once again, that a patient with signs and symptoms of an ARTI comes to you. After a thorough examination & laboratory investigation, you decided **NOT** to prescribe an antibiotic at this time. What kind of reaction would you expect from the patient? Do you think that such reactions from the patient could force the clinician to reconsider prescribing an antibiotic?

7) We are now close to finishing our discussion.

- In your opinion, what would you describe the current antibiotics prescription behavior of clinicians (including you yourself) while treating patients presenting with signs and symptoms of ARTIs? ***Probe****: appropriate prescription, over-prescription, under-prescription, …*
- *Can you elaborate a bit more …..*
- Can you tell me why clinicians might sometimes over-prescribe antibiotics to treat ARTIs?
- In your opinion, what would be the consequence(s) of antibiotic over-prescription or usage?

  Probe: economic burden? Resistance development?

- What measures you think should be put in place to reduce inappropriate antibiotic prescribing/usage in clinical practice?
- In your opinion, what kind of role clinicians could play to reduce inappropriate antibiotic prescription, and minimize the chance of drug resistance? What about the role of pharmacists/druggists? Would you after all say that they must have a role?

8) **We are almost finishing.**

- Can you tell me whether the clinic has a separate pharmacy/drug store to sell drugs to patient visitors?
- Do you currently have any working relationship with a pharmaceutical company or drug sale's representative? Can you tell me something more about this relationship (frequency of meeting etc …)?
- Can you tell me to which health professional association(s) you are affiliated with?
- In your opinion, would you say that antibiotic over usage is considered by the medical professional association you are affiliated with or by the Kenyan government as a serious public health/or medical problem?
- Finally, suppose the Kenyan government in collaboration with organizations (such as PharmAccess) brought in an intervention needed to reduce antibiotic over-prescription in clinical practice. In your opinion, what kind of reaction would you expect from clinicians on the ground? From patients?

Do you have any question for me?

This is the end, and thank you very much for participation.

B) Open-ended questions for patients

INTRODUCTION

Hello! My name is ……………… and I work for the PharmAccess Foundation, SafeCare/M-TIBA program in Nairobi, Kenya. (Jambo, kwa majina ninaitwa………. Na nafanya kazi na PharmAccess Foundation/M-Tiba hapa Nairobi)

I would like to have a short discussion with you about medication use during an acute respiratory tract infection. I will interview about 25 people in various communities around Nairobi. The results of this interview will be used to improve the quality of healthcare in these and other similar clinics here in Nairobi. (Ningependa kuzungumza nawe kuhusu matumizi ya dawa wakati unaugua na makali ya kupumua. Natarajia kuhoji watu 20 katika jamii mbalimbali humu Nairobi. Matokeo ya majibu haya zitatumika kuboresha huduma za afya katika hii kliniki na kliniki zingine.)

Let me assure you that the information you tell me now will only be used for healthcare quality improvement in the mTIBA clinics. Please, feel free to ask questions at any time during or after the interview. There is no right or wrong answer to these interview questions. If there are issues which you do not like to talk about, then let me know. (Nakuhakikishia ya kwamba taarifa utayonipa itatumika tu kuboresha huduma za afya katiki kliniki za M-Tiba. Ikiwa utakuwa na maswali wakati wowote wakati au baada ya mahojiano unaweza niuliza na kama kuna maswala ambayo hungependa kuzungumzia, nieleze)

Would you like to participate in this short interview? 1) Yes 2) No
(Ungependa kushiriki katika mahojiano haya? 1) Ndio 2) La/Hapana

Interviewer: If replied "YES", then thank the patient, ensure a written informed consent has been made, and start the interview.

1) Let's start by talking about your son's/daughter's current illness

- Can you, please, tell me what happened to your son/daughter? Which signs and symptoms of the illness led your son/daughter to seek a medical help today? How would you describe the severity of the illness? (Je unaweza nieleza nini kilichotokea na wewe? Ishara na dalili gani imesababisha uje hospitalini leo? Unaweza nieleza jinsi unavyo hisi?)

  Interviewer: please, check if the patient has mentioned any one of the following.

  - ☐ *Cough, (kohozi)*
  - ☐ *Fever (Increased Body T^o), (joto mwilini)*

☐ *Difficulty of Breathing, (ugumu unapo pumua)*

☐ *Fast Breathing, (kupumua kwa haraka)*

☐ *Nasal Congestion, (pua kufungana)*

☐ *Headache, (maumivu ya kichwa)*

☐ *Running Nose/Coryza, (mafua)*

☐ *Chest Pain, (maumivu ya kifua)*

☐ *Fatigue (Generalized Body Weakness),(kuchoka)*

☐ *Sore Throat, (mwasho wa koo)*

- So, can you tell me when the current signs and symptoms did start? (ulianza kuugua lini?)
- In your opinion, how would you say about the timing of your decision to bring your son/daughter for medical help today as compared to the time period during which these signs and symptoms start to manifest: is it EARLY or LATE? Why did you say EARLY? Would you have brought your son/daughter for a medical care today had you been responsible to cover all the health care expenses? (kwa maoni yako ungesema uamuzi wako wa kutembelea daktari saa hii ni kwa sababu maumivu yameanza ama yamezidi? Je ungelipia matibabu pekee yako ingekuwa hauna usiadizi wowote?)
- In response to the current health problem, did your son/daughter received any medical help elsewhere? Where specifically? Can you tell me if anyone of your relatives/friends/or neighbors advised you to start giving your son/daughter medications? What kind of medications did they tell you to start giving? Did they mention for you a place where to get these medications? (Ukikabiliana na kuugua, je ulitafuta usaidizi mahali pengine? Wapi haswa? Na majirani au marafiki au jamii yako ilikupa mawaidha kuhusu dawa ya kukusaidia? Walikwambia umeze au kunywa dawa gani? Na je walikufahamisha ni wapi unaweza pata dawa?)
- Then, have you already given your son/daughter medications by your own? What kind of medications? Where did you get access to these medications? (Umeshaanza kumeza dawa tayari? Dawa gani? Na ulipata dawa hizi wapi?)

2) Now, let's discuss about self-medication experiences

- Can you tell me if your son/daughter have had similar health problems in the past? (je unaweza nieleza kama umekuwa na matatizo ya afya kama sasa hapo awali?

- So, what do you do when similar health problems occur? (na matatizo ya afya yakitendeka unafanya nini?)
- Then, can you, please, elaborate your experiences with regard to antibiotics self-medication for similar kinds of health problems? (nieleze jinsi unavyohisi baada ya kumeza ‘’antibiotics’’)
- Do you have MEDICINES at home which are left over from a previous therapy? What do you do with these left-over medications? Where did you get access to these medicines? For which diseases? (unazo tembe za ‘’antibiotics’’ nyumbani ambazo hujameza ulipokuwa unaugua hapo awali? Unfanya nini na tembe hizi? Tembe hizo ulipata wapi?au ulinunua wapi?)
- Can you tell me your experience in regards to purchase of **OTC** drugs (i.e. without prescription) to treat similar signs and symptoms by your own? (nieleze unavyohisi unaponunua dawa bila daktari kukupa agizo?)

3) Let’s continue our discussion about medication prescription

- Can you show me whether the Doctor has prescribed to your son/daughter an antibiotic medication today? (Unaweza nionyesha kama daktari amekuagiza umeze ‘’antibiotics’’?) ***Interviewer: please, have a look at the prescription paper.***
- Can you tell me how did you get this medicine prescription from the doctor today? *Was it because you insisted on an antibiotic medication? What did you discuss with the Doctor about the need for an antibiotic medicine? (Ulipata dawa hii kivipi? Ulimueleza daktari ya kuwa unataka tembe za ‘’antibiotics’’? na mlizungumza nini kuhusu tembe hizi?)*
- *How did you tell the Doctor about your son’s/daughter’s current illness? Did you portray the severity of illness & emphasize the necessity of quick recovery? (Ulimueleza daktari nini haswa kuhusu kuugua kwako? Je ulimfahamisha ya kuwa maumivu imezidi na ungependa kupona kwa haraka?*
- When you came to the clinic today, what did you expect from the Doctor? Did you expect an antibiotic medicine today? Why? (Ulipokuja hospitalini ulikuwa unatarjia nini kutoka kwa daktari? Ulitarajia ya kuwa angekuagiza umeze tembe za ‘’antibiotic’’? kwanini?)
- In your opinion, would you say that similar signs and symptoms in the past did not get away without an antibiotic medicine, and thus your son/daughter definitely should get a medicine today? (kwa maoni yako ungesema ya kuwa ulipouugua hapo awali ilikuwa ni lazima upewe ‘’antibiotics’’ na kwa hivyo leo pia ungependa upewe ‘’antibiotics’’?)
- Suppose, the Doctor had decided **NOT** to prescribe for your son/daughter a medicine today because he thought the illness is NOT severe or will resolve by its own. Then, what would have been your reaction to this? What would you have said to the Doctor?

(Tuseme daktari akuelezee ya kuwa hatakupa tembe za ''antibiotics'' kwa sababu maumivu unayohisi haiyaitaji tembe hizo ungefanya nini? Ungemwambia daktari nini?)

- Now, let's assume that the Doctor decided to "wait and see" without prescribing a medicine for your son/daughter today, but told you to bring him/her back again if the disease gets worse. Then, would you agree to come back again (if the disease gets worse) or stay at home if your son/daughter feel better? (Na je daktari angekuambia usubiri kabla ya kukuagiza umeze tembe na akuagize urudi maumivu yakizidi ungekubali kurudi? Ama ungebaki nyumbani hadi upone?)

4) Finally, let's talk about payment for clinical care (Tuzungumze kuhusu jinsi unavyo lipia matibabu)

- Can you tell me who pays for your son's/daughter's health care costs today, including purchase of drugs? (Ni nani anayekulipia matibabu pamoja na dawa?)
  Probe*: You yourself, relatives, government, donor-money, …*
- In your opinion, how would you say about the affordability of health care costs, including the cost of drugs? (unaweza sema ya kuwa matibabu hapa Nairobi ni ya bei nafuu? Na bei ya dawa je?)
  Probe*: for clinic consultation, laboratory investigation, purchase of drugs, …*
- Imagine that you yourself were responsible to cover all the expenses related to your son's/daughter's health care today, including purchase of antibiotic medications. Then, would you still insist the Doctor to prescribe for you an antibiotic medication? What would you have done had the costs of antibiotics which the Doctor prescribed for your son/daughter today been expensive? (Tuseme ya kuwa ni wewe unashughulikia matibabu yako na ununuzi wa dawa. Ungependa daktari akuueleze dawa gani unafaa kumeza au la? Na ungefanya nini kama dawa ambayo daktari amekuagiza ununue ni ghali?)
- Can you explain to me your opinion about the side effects or consequences of using medicines without approval from a Doctor? (kwa maoni yako, unadhani kuna madhar aya kumeza dawa bila daktari kukuagiza umeze? Na ni madhara gani?)

Do you have any question for me you would like to ask? (Una swali yoyote ungependa kuniuliza?)
This is the end, and thank you very much for participation. (Asante sana. Tumefika mwisho wa mahojiano.)
